# Health-Related Quality of Life, Self-Efficacy and Enjoyment Keep the Socially Vulnerable Physically Active in Community-Based Physical Activity Programs: A Sequential Cohort Study

**DOI:** 10.1371/journal.pone.0150025

**Published:** 2016-02-24

**Authors:** Marion Herens, Evert Jan Bakker, Johan van Ophem, Annemarie Wagemakers, Maria Koelen

**Affiliations:** 1 Health and Society, Social Sciences Group, Wageningen University, Hollandseweg 1, PO Box 8130, 6700 EW, Wageningen, the Netherlands; 2 Mathematical and Statistical Methods, Plant Sciences Group, Wageningen University, Droevendaalsesteeg 1, PO Box 16, 6700 AA, Wageningen, the Netherlands; 3 Economics of Consumers and Households, Social Sciences Group, Wageningen University, Hollandseweg 1, PO Box 8130, 6700 EW, Wageningen, the Netherlands; Universidad Pablo de Olavide, Centro Andaluz de Biología del Desarrollo-CSIC, SPAIN

## Abstract

Physical inactivity is most commonly found in socially vulnerable groups. Dutch policies target these groups through community-based health-enhancing physical activity (CBHEPA) programs. As robust evidence on the effectiveness of this approach is limited, this study investigated whether CBHEPA programs contribute to an increase in and the maintenance of physical activity in socially vulnerable groups. In four successive cohorts, starting at a six-month interval, 268 participants from 19 groups were monitored for twelve months in seven CBHEPA programs. Data collection was based on repeated questionnaires. Socio-economic indicators, program participation and coping ability were measured at baseline. Physical activity, health-related quality of life and on-going program participation were measured three times. Self-efficacy and enjoyment were measured at baseline and at twelve months. Statistical analyses were based on a quasi-RCT design (independent t-tests), a comparison of participants and dropouts (Mann-Whitney test), and multilevel modelling to assess change in individual physical activity, including group level characteristics. Participants of CBHEPA programs are socially vulnerable in terms of low education (48.6%), low income (52.4%), non-Dutch origin (64.6%) and health-related quality of life outcomes. Physical activity levels were not below the Dutch average. No increase in physical activity levels over time was observed. The multilevel models showed significant positive associations between health-related quality of life, self-efficacy and enjoyment, and leisure-time physical activity over time. Short CBHEPA programs (10–13 weeks) with multiple trainers and gender-homogeneous groups were associated with lower physical activity levels over time. At twelve months, dropouts' leisure-time physical activity levels were significantly lower compared to continuing participants, as were health-related quality of life, self-efficacy and enjoyment outcomes. BMI and care consumption scored significantly higher among dropouts. In conclusion, Dutch CBHEPA programs reach socially vulnerable, but not necessarily inactive, groups in terms of socio-economic and health-related quality of life outcomes. Our findings suggest that CBHEPA programs particularly contribute to physical activity maintenance in socially vulnerable groups, rather than to an increase in physical activity behaviour over time.

## Background

Physical inactivity has been identified by the WHO as the fourth leading risk factor for global mortality [1, 2]. Health disorders associated with inactivity, including impaired health-related quality of life, as well as direct and indirect economic costs, impose a substantial burden on societies and health systems [3]. In the Netherlands, socially vulnerable groups, e.g., those with low socio-economic status (SES) or of non-Dutch origin, are less engaged in sport and physical activity (PA) than high SES groups [4, 5]. Over the past decade, Dutch policy has been to promote community-based health-enhancing physical activity (CBHEPA) programs in order to improve physical activity behaviour and health-related quality of life, in particular targeting socially vulnerable groups [6, 7].

The relationship between PA behaviour and health-related quality of life is, however, a rather complex one. Demographic factors, as well as biological, psychosocial, behavioural, social and cultural factors, influence this relationship [2, 8, 9]. CBHEPA programs aim to change individual PA behaviour and to enhance PA maintenance and program adherence, using concepts such as attitude, subjective norms, self-efficacy [10, 11], social support [12, 13] and PA enjoyment [14, 15]. The need to address interpersonal aspects alongside individual approaches is widely recognised in PA promotion [16, 17]. Consequently, the theoretical grounds of CBHEPA programs are based on an ecological perspective on human health [18, 19]. The ecological perspective emphasises the need to take into consideration interaction between factors within and across different levels, such as individual, group and community level [20, 21].

### Evaluating the effectiveness of CBHEPA programs

The ecological perspective used in CBHEPA programs, as well as differences described in the literature between PA initiation and PA maintenance [22], pose several challenges to evaluating the effectiveness of CBHEPA programs. Firstly, most research on the explanatory variables and correlates of PA behaviour has focused on individual level factors [2]. The multiple levels addressed by CBHEPA programs require a multilevel approach to hypothesis testing, taking into account the interdependencies within and between individuals, groups and communities [18, 19, 21, 23–25]. Secondly, Dutch CBHEPA programs often target specific societal groups within a community, such as the socially vulnerable. Identifying indicators and instruments suitable to measure PA behaviour and health-related quality of life in these groups is a challenge [26]. Thirdly, alongside measurement issues, recent literature indicates that factors predicting initial change in PA behaviour differ from those predicting PA maintenance [22, 27–30]. So far, no uniform standards are in use to define PA maintenance [31]. A commonly used definition is being physically active once a week for a period of at least six months [32]. Some studies indicate that factors relevant for PA behaviour initiation are best defined in terms of pre-motivational and motivation factors, such as awareness, knowledge and (health) risk perception, attitude, self-efficacy and social influence [22]. In PA maintenance, post-motivational factors, i.e. psychological constructs bridging the gap between intention and behaviour, such as self-regulatory processes, the ability to cope with stressors in daily life [33, 34] and so-called maintenance self-efficacy, are factors of importance [22, 27, 35, 36]. In addition, PA enjoyment is found to be a moderator of self-efficacy in PA behaviour [17]. Studies indicate that not only self-control and discipline, but also enjoyment, pleasure and ‘not worrying’, are key values in maintaining an active and healthy lifestyle [14, 15, 37]. Fourthly, evaluating CBHEPA programs requires group effects to be taken into consideration. Several studies illustrate the importance of group support and group dynamics for the effectiveness of (CBHE)PA programs. Group dynamics in CBHEPA programs are, however, often implicit and not accounted for. CBHEPA programs are usually group-based for organisational reasons (cost-covering), rather than for behavioural change reasons [38]. Nevertheless, some studies indicate that group dynamics strategies, explicitly applied in group-based PA interventions, are more effective in establishing change in PA behaviour than individually targeted interventions with social support, which, in turn, are more effective than individual interventions without additional social support [39, 40].

Although many strategies have been developed to increase PA levels [41, 42], affect sizes are usually small to moderate [2]. Most evidence is built on correlational, cross-sectional studies at participant level, lacking insight into causal relationships between factors influencing PA [2, 41, 43]. Longitudinal designs including time varying determinants of PA behaviour and maintenance are rare [18]. In view of the aims of Dutch group-based CBHEPA programs, our study focuses on evaluating participants’ PA behaviour and maintenance in relation to multilevel explanatory factors and time varying covariates. With a sequential cohort study, we aim to contribute to the evidence-base of CBHEPA programs and their potential to increase and sustain PA levels and health-related quality of life in inactive, socially vulnerable people. The advantage of a sequential cohort design, monitoring CBHEPA program participants for a specified period of time, is that simultaneously multiple (intermediate) outcomes can be studied over a period of time and can increase the power of the statistical procedures used to determine whether a change has taken place. It allows us to control for possible history and maturity effects [44]. Consequently, to measure effects, a sequential cohort design is a promising alternative to a randomised controlled trial (RCT) design, which is considered less appropriate to assess the effectiveness of CBHEPA programs [45, 46]. In this paper, we address the question: *Do CBHEPA programs contribute to an increase and maintenance of physical activity in socially vulnerable groups over time*?

## Methods

To assess the outcomes of CBHEPA programs at participant level, we examined on-going Dutch CBHEPA programs, summarised under the denominator ‘Communities on the Move’ (CoM). CoM was developed and disseminated by the Netherlands Institute for Sports and PA (NISB) from 2003 to 2012. CoM targets inactive, socially vulnerable groups with the aim of enhancing PA levels, hence contributing to participants’ health-related quality of life. Since 2012, CoM has been subject to a comprehensive evaluation study, including assessment of its effectiveness at participant level [21].

### Study population

Participants from 19 groups (10–20 participants) were recruited in on-going CBHEPA programs targeting socially vulnerable groups in seven different municipalities. Local CBHEPA program representatives were approached through the NISB network, information meetings, training sessions, field visits and snowball procedures (Table 1). This resulted in access to one or more groups per CBHEPA program. Recruitment of participants within groups was based on a non-randomised, purposive sampling approach. Participation was on a voluntary basis.

**Table 1:** Overview of CBHEPA programs included.

CBHEPA program	Municipality	Target group	Program design	Group composition	# groups	# participants
1	Amsterdam	▪Socially vulnerable	▪Fixed duration (10 weeks)	Women	1	14
		▪Non-Dutch origin	▪Outdoor			
			▪Walking/running			
			▪Once a week			
			▪multiple exercise trainers			
2	Den Haag	▪Socially vulnerable	▪Continuing	Women	3	31
		▪Non-Dutch origin	▪In-/outdoor			
			▪Exercise to music/fall prevention/walking			
			▪Once a week			
			▪One known exercise trainer			
3	Enschede	▪Socially vulnerable	▪Fixed duration (13 weeks + 18 months follow-up meetings every 6 weeks)	Women	2	30
		▪Dutch and non-Dutch origin	▪In-/outdoor	Men	1	
			▪Mixed sports activities			
			▪Once a week			
			▪Multiple exercise trainers			
4	Helmond	▪Socially vulnerable	▪Continuing	Mixed	2	39
		▪Dutch and non-Dutch origin	▪Outdoor			
			▪Outdoor fitness			
			▪Multiple times a week			
			▪One known exercise trainer			
5	Hengelo	▪Socially vulnerable elderly	▪Fixed duration (12 weeks)	Women	3	51
		▪Dutch and non-Dutch origin	▪In-/outdoor	Men	1	
			▪Mixed sports activities			
			▪Once a week			
			▪Multiple exercise trainers			
6	Rotterdam	▪Socially vulnerable and elderly	▪Continuing	Women	3	73
		▪Mostly non-Dutch, some Dutch origin	▪Indoor	Men	1	
			▪Exercise to music/fall prevention			
			▪Multiple times a week			
			▪One known exercise trainer			
7	Tilburg	▪Socially vulnerable, chronically ill elderly	▪Continuing	Women	1	30
		▪Dutch origin	▪Indoor	Mixed	1	
			▪Fall prevention exercises/mixed sports activities			
			▪Once a week			
			▪One known exercise trainer			

A total of 268 participants was included at baseline, mostly women (86.7%). Personal and socio-economic indicators showed that mainly middle-aged participants (mean age 58.6 years; *sd*: 14.0) of non-Dutch origin (64.6%),were involved. Furthermore, participants were low (48.6%) to moderately (42.4%) educated and a substantial proportion (52.4.7%) had low incomes (<€1,350/month). A minority (11.6%) had a full- or part-time job, 16.9% lived on income support (social benefit), and one fifth (20.6%) were retired. Nearly one third (29.2%) were single households, one third (30.0%) lived with a partner and a little over one third (39.6%) with a partner and/or children (Table 2).

**Table 2:** Participants’ personal and socio-economic characteristics (n = 268).

Variable	N	%	Mean *(sd)*
***Personal characteristics***			
Gender			
Women	229	86.7	
Men	35	13.3	
Age			
< 50 years	78	31.2	
50–64 years	92	36.8	
65–74 years	52	20.8	
> 75 years	28	11.2	
	250		58.6 *(14*.*0)*
Ethnic origin (n = 263)			
Dutch	93	35.4	
Non-Dutch*	170	64.6	
***Socio-economic characteristics***			
Education (n = 256)			
No/primary education	124	48.6	
Secondary education	109	42.4	
College/university education	23	9.0	
Household income			
< € 1,000	65	25.4	
€1,001–€1,350	69	27.0	
€1,351–€1,800	30	11.7	
> €1,801	20	7.8	
Income not specified	72	28.1	
Employment status			
Working full-/part-time	31	11.6	
Job seeking	32	12.0	
Incapacity for work	18	6.7	
Income support	45	16.9	
Retired	55	20.6	
Household conditions			
Single	76	29.2	
With partner	78	30.0	
With partner and/or child(ren)	103	39.6	
other	3	1.2	

* Number of countries of origin: 29

### Data collection

**Fig 1:**
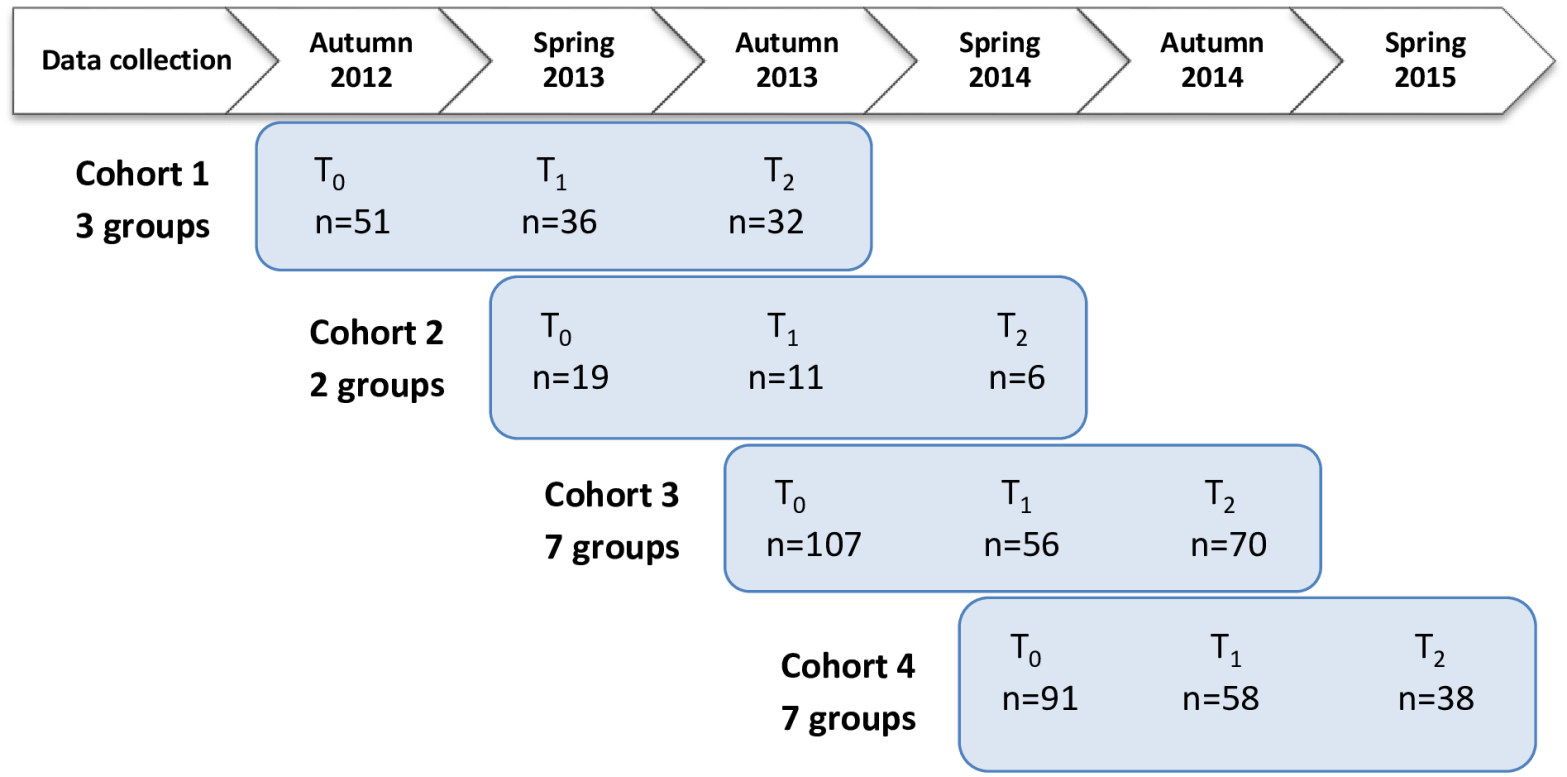
Data collection procedure.

Our study was based on a sequential cohort design. Participants were recruited and monitored in four sequential cohorts. Data collection for cohort 1 started in autumn 2012, and for cohort 4 in spring 2014. In order to reach the generally hard-to-reach socially vulnerable groups [47], we applied a personalised approach, reaching out to gatekeepers, such as the exercise trainer, and making ourselves known to CBHEPA participants. Data were collected by a researcher (first author) and a group of trained assistants at three points in time: T_0_, T_1_ at six months and T_2_ at twelve months (Fig 1).

Questionnaires were developed based on validated survey instruments available for the Dutch population. Thus, we tried to select instruments most appropriate for the socially vulnerable target group. Socio-economic indicators, program participation and sense of coherence to assess coping ability were measured at baseline. Data on socio-economic indicators (age, income, education, employment status, living conditions) were collected in accordance with standardised questions of the Local and National Monitor Public Health in the Netherlands [48, 49]. Data on individual motivations to participate in the CBHEPA program were collected using an open-ended question. Data on past and present sport and PA behaviour were collected, assessing program participation time prior to baseline measurement and (former) sports club membership. People’s ability to cope with stressors in daily life was measured using the SoC three-item, three-point scale for sense of coherence [50–53]. Questions were: *Do you usually see solutions to problems and difficulties that other people find hopeless* (manageability)? *Do you usually feel that your daily life is a source of personal satisfaction* (meaningfulness)? And: *Do you usually feel that the things that happen to you in your daily life are hard to understand* (comprehensibility)?

PA behaviour, health-related quality of life and on-going program participation were measured three times. PA and sport behaviour were measured using the validated Short Questionnaire for Sport and Physical Activity (SQUASH), measuring self-reported work-related, domestic, leisure-time and sport-related physical activities in minutes per week [54, 55]. The SQUASH generates data that can be compared with national and regional data, as Dutch trend analyses for PA behaviour over the past two decades are based on the SQUASH, offering a vast body of reference data for our study [5].

Health-related quality of life data were repeatedly measured at all three time points using two indicators: the five-dimension, three-level descriptive Euro Quality of Life questionnaire (EQ-5D-3L), assessing self-reported levels of complaints on ‘mobility’, ‘self-care’, daily activity’, ‘pain’ and ‘anxiety’ [56, 57]. Based on the outcomes of the EQ-5D-3L, the EQ-Index (ranging from -1 to 1) was computed, defining a ‘health state’ using the Dutch time-trade-off value set [58, 59]. Perceived health was measured using a visual analogue scale (EQ-VAS), ranging from 0 to 100 [56]. EQ-VAS measures how participants perceive their health at a particular point in time [59].

PA self-efficacy and PA enjoyment were measured at baseline and at the last measurement (T_2_). PA self-efficacy was measured using a five-item, five-point scale [60]. Statements were: *I am confident that I am able to continue to participate in the PA program during the coming months*, and *I am confident that I am able to continue to participate in the PA program when I am tired*. PA enjoyment was measured using a nine-item, five-point scale, translated and adapted from the Physical Activity Enjoyment Scale [61]. Statements were: *When I do exercise or sports*, *I enjoy it*, and *When I do exercise or sports*, *is it fun to do*, or *When I do exercise or sports*, *I feel bored*.

In the supporting information (S1 Table) an overview is presented of variables measured over time in relation to PA behaviour.

At each measurement, questionnaires were individually completed by participants during or after a group training session at the sports venue. Informed consent was arranged orally on the spot and confirmed in writing for each respondent. The researcher explained the purpose of the study at each session. Both the researcher and trained assistants helped respondents who had difficulty filling out the questionnaire by giving instructions or by adopting an interview style. The number of assistants varied with group composition: from one for groups with only Dutch native speakers to a maximum of five in groups with migrant respondents. Dutch was the working language, since ethnic diversity within groups was large (>10 countries of origin). Interpretation, if needed, was provided by an assistant or a Dutch speaking fellow group member from a similar background. Completion of the baseline questionnaire took on average 35–40 minutes, and of the follow-up questionnaires on average 20–25 minutes. After filling out the questionnaire, respondents were treated to fruit snacks and drinks.

Follow-up rate for all four cohorts at T_1_ was 60% (n = 161). In response to these follow-up rates, additional data collection strategies were initiated during the third year (2014). Participants and ex-participants were contacted in places where they habitually assembled, usually a community centre. Follow-up questionnaires were sent to home addresses, accompanied if possible by a telephonic reminder after two weeks. Overall follow-up rate at T_2_ was 55% (n = 146), showing a 91% recovery rate of T_1_ participants.

Reasons for program dropout were either personal (health issues or life events) or program related (program activities ceased to exist). Reasons for not being willing to participate in follow- up measurements, given in 5% of cases, were: reluctance to fill out questionnaires in general, not being able to fill out the questionnaire by themselves, doubt about the relevance of the questions, and sometimes people told the researchers that there was no need, since ‘nothing changes anyway’.

Information about the organisation of the CHEPA program and group composition was collected during each session by the researcher and assistants, reported in observational notes. Thus, information was gathered about the measurements, e.g., difficulties in understanding questions or concepts, as well as additional information on group developments and participants.

### Data analysis

In order to investigate the effectiveness of CBHEPA programs comprehensively, addressing the question whether CBHEPA programs contribute to an increase in and maintenance of physical activity in socially vulnerable groups, we tested three hypotheses using a combination of statistical procedures (SPSS22). Alongside significance, effect sizes (Cohen’s d and Pearson’s *r*) were reported for the main outcomes of interest.

First, based on a rather traditional approach, we compared groups who participated for a year with groups which had just started. The hypothesis was: Participation in a CBHEPA program for one year leads to higher PA levels and health-related quality of life outcomes in its participants compared to starters (H1). A quasi-randomised control trial (RCT) design was used to measure change in PA behaviour and health-related quality of life outcomes between groups. The T_0_ comparability of the different cohorts was first tested. Then baseline group means of cohort 4 (nine groups; n = 91), treated as ‘control group by proxy’, were compared with T_2_ group means after twelve months for cohorts 1 and 2 (four groups; n = 38), using an independent *t*-test. It was decided to compare group means using independent *t*-tests to take into account the interdependency of observations within PA groups. Cohort 3 was not included in this analysis since the measurements overlapped with measurements in cohorts 1 and 2.

Second, we compared participants who remained active in the CBHEPA programs with those who were no longer active (‘program dropouts’). The hypothesis was: CBHEPA participants perform better on physical activity and health-related quality of life outcomes than participants who dropped out of the CBHEPA program (H2). The Mann—Whitney U test was used to compare PA levels and health-related quality of life outcomes.

Third. since these types of analysis still did not provide for deeper insights in the main question whether CBHEPA programs contribute to an increase in and maintenance of physical activity in socially vulnerable groups over time, we developed an integrated multilevel model. The hypothesis was: Participation in a CBHEPA program leads to increase in and maintenance pf its participants’ daily physical activity levels over time (H3). A longitudinal multilevel analysis was used to examine the growth model of PA levels over time. As a result of our data collection strategy, our dataset was characterised by intra-individual interdependencies in the repeated measurements, as well as inter-individual interdependencies in the group wise measurements. Therefore, multilevel modelling was used because it is less sensitive to absence of normality in the data and lack of independent sampling of participants and observations. It takes into account group interdependencies, which are considered of importance for effectiveness in CBHEPA programs [44, 62]. Another advantage of multilevel analysis of longitudinal data is its ability to handle missing data [63]. This includes the ability to handle models with varying measurement occasions [64, 65]. Unlike fixed occasion models, for example MANOVA, multilevel regression models do not assume equal numbers of observations, or fixed measurement occasions, so respondents with missing observations pose no special problems, and all cases can remain in the analysis. This is an advantage, because larger samples increase the precision of the estimates and the power of the statistical tests [44]. To deal with missingness, in our study we assumed data to be data missing at random (MAR), a indicating that the missingness may depend on other variables in the model, and through these be correlated with the unobserved values [44].

For our data, three levels were defined: intrapersonal, estimating variance of repeated measurements within individuals; interpersonal, estimating variance of fixed factors between individuals; and group level, estimating variance between groups (Table 3). Leisure-time physical activity (LTPA) was used as primary outcome indicator, since the CBHEPA programs included in our study offered leisure-time PA schemes. We therefore assumed that LTPA was a more sensitive indicator for change than overall PA behaviour. Since the outcome of LTPA was not normally distributed, we used a log transformed LTPA variable (LOG LTPA).

**Table 3:** Data definition for multilevel longitudinal analysis of PA behaviour.

Variable	Level	Description	Values	Measurement
***General***				
Time of measurement	Within individual	Variable representing three linear occasions (at 6-month intervals) of measurement	1 = Measurement T_0_	Scale
			2 = Measurement T_1_	
			3 = Measurement T_2_	
Participation in CBHEPA program	Between individual	Variable, identifying on-going CBHEPA participation or not	0 = no; 1 = yes	Nominal
***Personal and socio-economic***				
Resp	Between individual	A within group identifier representing each respondent (id, group, cohort)	11001 to 194010	Ordinal
Age	Between individual	Predictor variable, classifying age groups	1 = < 50 years	Ordinal
			2 = 50–64 years	
			3 = 65–74 years	
			4 = ≥75 years	
Gender	Between individual	Predictor variable, identifying gender	0 = women; 1 = men	Nominal
Ethnic origin	Between individual	Predictor variable, identifying Dutch versus non-Dutch respondents	0 = no; 1 = yes	Nominal
Education low	Between individual	Predictor variable, identifying low versus not low educational level	0 = no; 1 = yes	Nominal
***Health-related quality of life***				
EQ index	Within individual	Predictor and outcome variable EuroQoL5D-3L, describing severity of complaints (mobility, pain, daily activities, anxiety)	-1–1	Scale
EQ-VAS	Within individual	Predictor and outcome variable, visual analogue scale representing perceived health	0–100	Scale
Tot. SoC	Between individual	Predictor variable, measuring sense of coherence (coping capacity)	3–9	Scale
***Sport and physical activity***				
LOG Tot LTPA	Within individual	Outcome variable (log transformed) measuring self-reported leisure-time PA behaviour, including sport and CBHEPA participation (minutes/week)	0.00–3.72	Scale
LOG Tot PA	Within individual	Outcome variable (Log Transformed) measuring total PA behaviour (minutes/week)	1.49–3.97	Scale
PA self-efficacy	Within individual	Predictor variable, 5-item scale measuring PA self-efficacy, using 5-point scale (fully disagree to fully agree)	5–25	Scale
PA enjoyment	Within individual	Predictor variable, 9-item scale measuring PA enjoyment, using 5-point scale (fully disagree to fully agree)	9–45	Scale
***Group***				
BG	Group	Group identifier variable	1–19	Ordinal
BG_type	Group	Variable identifying group characteristics in terms of program duration, trainer and group composition (men/women)	1 = fixed, multiple trainers, homogeneous	Nominal
			2 = fixed, single trainer, homogeneous	
			3 = continuing, single trainer, homogeneous	
			4 = continuing, single trainer, heterogeneous	

**Fig 2:**
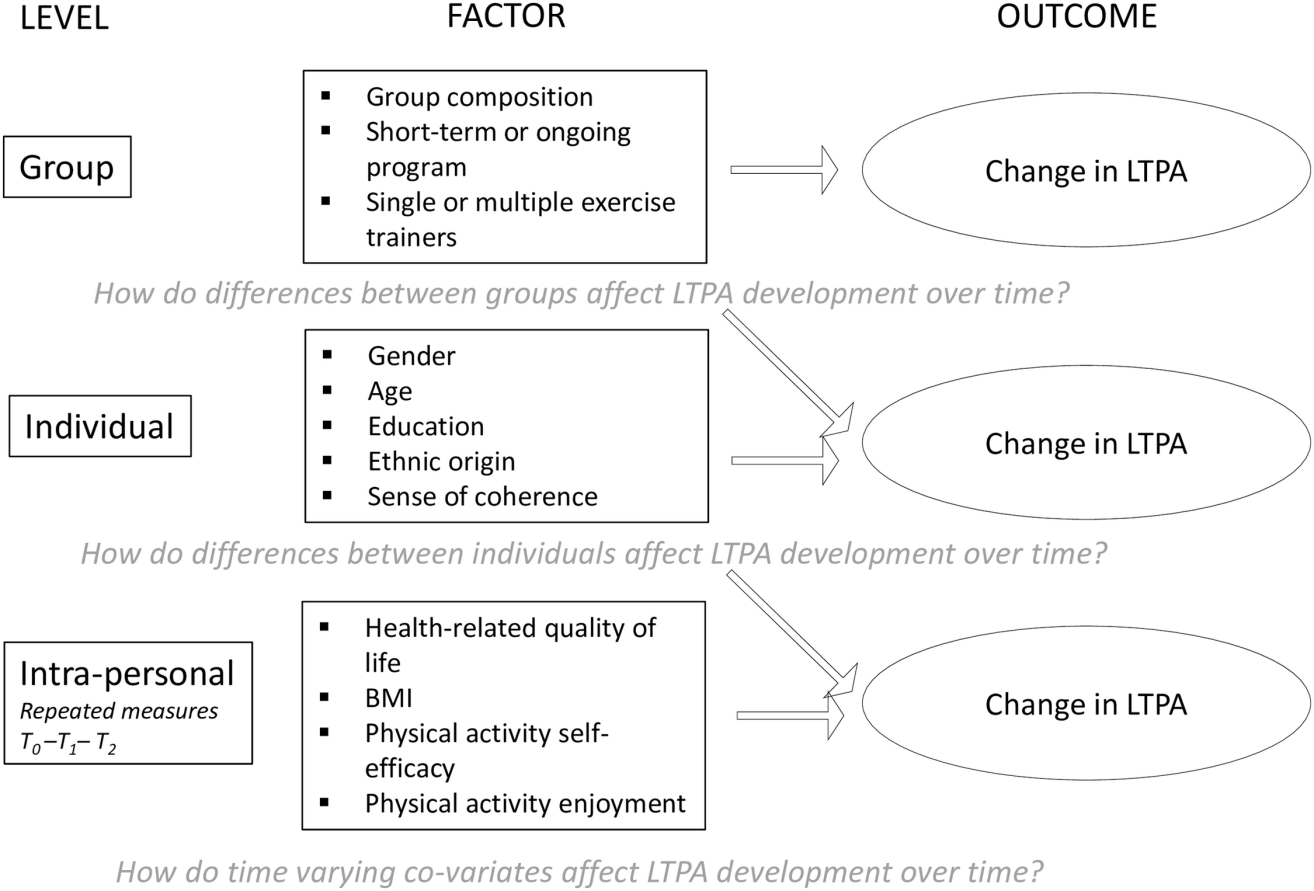
Multilevel perspective on change in LTPA through CBHEPA programs (after Heck et al. [66]).

Three-level regressions models were developed to assess change over time in LTPA (minutes/week) (Fig 2).

Forward multilevel modelling was used [62], starting with a null model based on LOG LTPA as outcome indicator, time (repeated measurements) and program participation. Interaction terms for time and program participation were included. Then stepwise fixed factors, such as gender, age, ethnic origin, educational level and program participation time were included, as well as SoC (coping ability), followed by time varying covariates for health-related quality of life, BMI, PA self-efficacy and PA enjoyment. Model estimation was based on the restricted maximum likelihood (REML). REML estimates the variance components after removing the fixed effects from the model. REML estimates have less bias than full maximum likelihood estimates, are more realistic and therefore thought to be more suitable when the number of groups is small [44]. As we were dealing with repeated measurements, we used the autoregressive structure (AR(1)) as first order covariance structure. For random effects, we used the scaled identity covariance structure [66]. The group level was defined as first level, since participants are nested within groups; the participants were defined as second level and the repeated measurements as third level. Parallel multilevel modelling procedures were conducted, taking into consideration two different indicators for health-related quality of life: one for perceived health (EQ-VAS) and one for self-reported levels of health problems (EQ-Index). An example of the syntax developed for multilevel modelling in SPSS 22 is presented in the supporting information (S1 Text).

The authors declare that the study was conducted in accordance with general ethical guidelines for behavioural and social research in the Netherlands, peer-reviewed and approved by the review board of the Wageningen School of Social Sciences. Guarantees of anonymity were given prior to each round of data collection. Participants were able to withdraw from the study at any time for any reason.

## Results

Baseline health-related quality of life outcomes showed a mean EQ-Index score of 0.72 (*sd*: 0.28). The majority of participants reported pain-related health complaints (69.2%). Mean perceived health (EQ-VAS) scored 70.24 (*sd*: 15.74). Mean BMI scored 29.52 (*sd*: 5.85). The majority (67.0%) had paid a visit to a care professional during the four weeks prior to the baseline measurement. Mean SoC (Cronbach’s α = 0.43) scored 6.98 (*sd*: 1.33). Respondents’ SoC-scores were categorised into people with a high SoC (14.3%), a moderate SoC (51.2%) and a weak SoC (34.4%).

Baseline sport and PA outcomes showed that mean overall PA level scored 1513 minutes/week (*sd*: 1094). Most time was spent on household PA, on average 778.6 minutes/week (*sd*: 848.3). Many participants (83.4%) were involved in LTPA (e.g., walking, cycling and gardening) at baseline, on average 355 minutes/week (*sd*: 473). Fewer participants (43.3%) were involved in sports, on average 70.8 minutes/week (*sd*: 140.4). The majority were not members of a sports club (75.9%). Prior to the baseline inquiry, over half of the participants (52.2%) had participated for less than three months in the CBHEPA program, 15.3% between three and six months, and 32.5% longer than six months. The majority (68.9%) participated once a week, 28.5% more than once a week and 2.6% less than once a week. Mean PA self-efficacy (scale 5–25; Cronbach’s α = 0.70) scored relatively highly: 20.12 (*sd*: 3.97). Mean PA enjoyment (scale 9–45; Cronbach’s α = 0.73) scored also relatively highly: 39.9 (*sd*: 6.1) (Table 4).

**Table 4:** Baseline health-related and PA outcomes for participants.

Variable	N	%	Mean *(sd)*
***Health-related Quality of Life***			
EuroQoL 5D-3L *(% reporting complaints*)			
Walking	101	38.5	
Self-care	28	10.7	
Daily activities	102	38.6	
Pain	178	69.2	
Anxiety	91	34.4	
EQ-Index (*scale -1–1*)	260		0.72 (*0*.*28)*
EQ-VAS (*scale 0–100*)	259		70.24 *(15*.*74)*
BMI (n = 250)	250		29.52 *(5*.*85)*
Contact health professional *(past 4 weeks)*			
Yes	179	67.0	
No	88	33.0	
Sense of coherence *(scale 3–9)*			
Strong SoC (score 9)	35	14.3	
Moderate SoC (score 8*–*7)	125	51.2	
Weak SoC (score 6*–*3)	84	34.4	
	244		6.98 *(1*.*33)*
***Sport and physical activity***			
Commuting PA *(min/week)*	268		40.2 (*125*.*3);* 0
Work-related PA *(min/week)*	268		181.5 *(483*.*9)*
Household-related PA *(min/week)*	268		778.6 *(848*.*3)*
Leisure-time PA (LTPA) *(min/week)*	268		355.1 *(472*.*5)*
Sport *(min/week)*	268		70.8 *(140*.*4)*
Total LTPA, incl. CBHEPA and sport *(min/week)*	268		507.8 *(517*.*6)*
Total PA *(min/week)*	268		1513.1*(1093*.*8)*
PA self-efficacy scale	242		20.12 *(3*.*97)*
PA enjoyment scale	239		39.9 *(6*.*1)*
Program participation at baseline			
< 3 months	130	52.2	
3–6 months	38	15.3	
> 6 months	81	32.5	
Frequency program participation			
< 1 x week	7	2.6	
1 x week	184	68.9	
2 x week	51	19.1	
> 2 x week	25	9.4	
(Former) Sports club member			
Yes	59	24.1	
Former sport member	86	35.1	
No, never	100	40.8	

**Fig 3:**
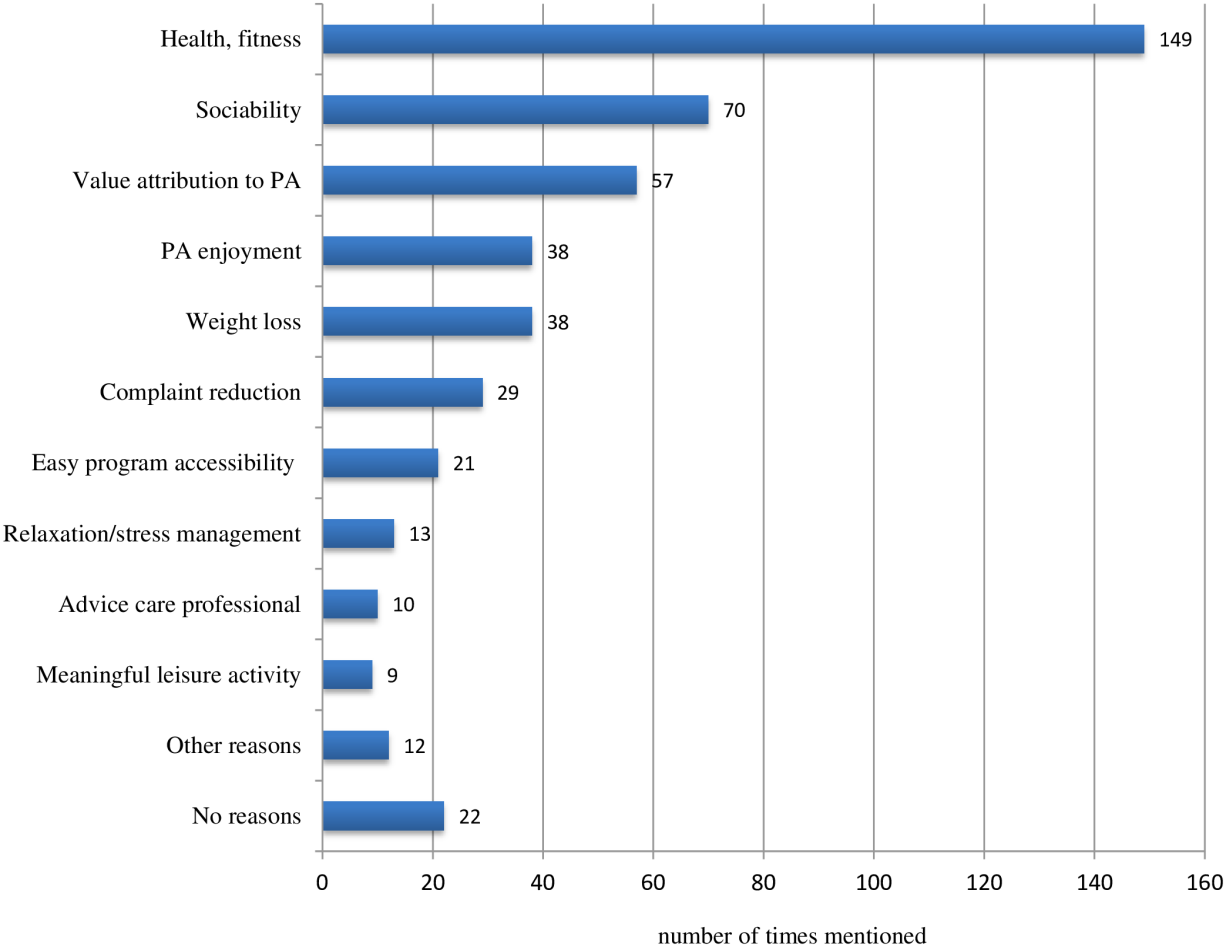
Self-reported participant motivations for joining CBHEPA programs (n = 268).

Individual motivations to join a CBHEPA program were mostly health and physical fitness, followed by sociability, value attribution to physical activity, enjoying physical activity and weight loss. Participants often reported more than one motivation (Fig 3).

### Measuring effectiveness using a ‘control group by proxy’

At baseline, no significant differences were found between cohorts 1, 2 (four groups; n = 70) and cohort 4 (nine groups; n = 91) for gender, age, income, and low and moderate educational levels (*z*-approximation of Mann–Whitney U test). High educational levels were significantly found more in groups of cohort 4 (*z* = 2.27, *p* = 0.024). For PA levels, no significant differences (*t*-test) were found between cohorts 1, 2 and 4 for baseline group means LOG LTPA (*t*(11): -0.04, *p* = 0.97) and for group means (log transformed) total PA behaviour (*t*(11)-0.42, *p* = 0.68) (Table 5). For health-related quality of life, no significant differences were found between cohorts 1, 2 and 4 in baseline group means for EQ-Index, EQ-VAS and BMI, indicating comparability in health-related conditions between the groups. Also, no significant differences were found between cohorts 1, 2 and 4 in baseline group means SoC scores and group means PA self-efficacy scores. For PA enjoyment, baseline group means scores were significantly lower in cohort 4 than in cohorts 1 and 2 (Table 5). The effect size (Cohen’s d) was 1.5, indicating a large difference in self-reported PA enjoyment between the cohorts at baseline.

**Table 5:** Group means comparison for cohorts 1, 2 and 4 at baseline (T_0_) and at twelve months (T_2_).

Variable	Cohort comparison	*M*	*SE*	*t*	*df*	*p*	Cohort comparison	*M*	*SE*	*t*	*df*	*p*
***Health-related Quality of life***												
EQ-Index	T_0_ Cohort 1,2	0.75	0.04	-0.42	11	0.68	T_2_ Cohort 1,2	0.83	0.03	-1.31	11	0.22
	T_0_ Cohort 4	0.71	0.06				T_0_ Cohort 4	0.71	0.06			
EQ-VAS	T_0_ Cohort 1,2	71.84	2.50	-0.54	11	0.60	T_2_ Cohort 1,2	72.90	3.52	-0.71	11	0.49
	T_0_ Cohort 4	68.99	3.29				T_0_ Cohort 4	68.99	3.29			
BMI	T_0_ Cohort 1,2	29.30	0.77	0.09	11	0.93	T_2_ Cohort 1,2	27.68	0.54	1.62	11	0.13
	T_0_ Cohort 4	29.40	0.66				T_0_ Cohort 4	29.40	0.66			
***Sport and physical activity***												
Total leisure-time PA (LOG)	T_0_ Cohort 1,2 ^a^	2.60	0.08	-0.04	11	0.97	T_2_ Cohort 1,2 ^c^	2.47	0.09	1.14	11	0.28
	T_0_ Cohort 4 ^b^	2.60	0.07				T_0_ Cohort 4	2.60	0.07			
Total PA (LOG)	T_0_ Cohort 1,2	3.07	0.03	-0.42	11	0.68	T_2_ Cohort 1,2	3.09	0.07	-0.57	11	0.58
	T_0_ Cohort 4	3.03	0.07				T_0_ Cohort 4	3.03	0.07			
PA self-efficacy	T_0_ Cohort 1,2	20.58	2.58	-0.22	11	0.84	T_2_ Cohort 1,2	18.94	1.29	0.77	11	0.46
	T_0_ Cohort 4	20.13	0.86				T_0_ Cohort 4	20.13	0.86			
PA enjoyment	T_0_ Cohort 1,2	32.52	4.21	-2.50	11	0.03*	T_2_ Cohort 1,2	37.69	3.00	-4.85	11	0.001**
	T_0_ Cohort 4	24.19	1.34				T_0_ Cohort 4	24.19	1.33			

^a^ T_0_ Cohort 1, 2 (4 groups, n = 70);

^b^ T_0_ Cohort 4 (9 groups; n = 91);

^c^ T_2_ Cohort 1, 2 (4 groups, n = 38);

*p<0.050;

** *p*<0.001

To measure the effectiveness of CBHEPA programs, the next step was to compare T_2_ group means—measured after twelve months—of cohorts 1 and 2 (4 groups; n = 38) with baseline group means of cohort 4 (9 groups; n = 91) for PA and health-related quality of life outcomes (*t*-test). No significant differences were found between the ‘active’ and ‘control group by proxy’ for LOG LTPA (*t*(11) 1.14, *p* = 0.28) and (log transformed) total PA (*t*(11) -0.57, *p* = 0.58). Also, no significant differences were found for the health-related quality of life indicators EQ-Index, EQ-VAS, BMI and PA self-efficacy. For PA enjoyment, the T_2_ group means scores were significantly higher after twelve months among the ‘active’ participants than in the groups just starting (*t*(11) -4.85, *p* = 0.001) (Table 5). The effect size (Cohen’s d) was 2.9, nearly double the effect size at baseline, indicating a large effect.

The dataset used for the groups means comparison can be found in the supporting information (S1 Dataset).

We did not find evidence to support hypothesis (H1) that participation in a CBHEPA program for one year leads to higher physical activity levels and health-related quality of life among its participants compared to a starting control group. We did find, however, significant differences in PA enjoyment scores between groups in cohorts 1, 2 and 4 at baseline as well as at T_2_.

### CBHEPA participants versus program dropouts

Over the course of six months, between group comparisons showed that program dropouts scored significantly lower for LTPA in minutes/week (*z* = 1.99, *p* = 0.047) and perceived health status (EQ-VAS; *z* = 2.88, *p* = 0.004). No between group differences were found for overall PA, EQ–Index, BMI and contact with care professionals (Table 6).

**Table 6:** Differences between participants (part) and program dropouts (pdo) in PA behaviour and health-related quality of life at T_1_ and T_2_ (*z-*approximation of Mann–Whitney U test).

	T_1_							T_2_						
Variable	N total	N part	N pdo	Test stat	*z*	*p*	r^a^	N total	N part	N pdo	Test stat	*z*	*p*	r^a^
***Health-related quality of life***														
EQ-Index	154	138	16	1261.5	0.95	0.343	0.08	141	117	24	1773.0	2.07	0.039*	0.17
EQ VAS	151	134	17	1620.5	2.88	0.004**	0.23	140	117	23	1683.5	1.93	0.053	0.16
BMI	142	128	14	782.0	-0.78	0.435	-0.70	135	113	22	879.0	-2.17	0.030*	-0.19
Contact care professional	156	139	17	910.0	-1.80	0.073	-0.15	144	120	24	1080.0	-2.24	0.025*	-0.19
***Sport and physical activity***														
Total leisure-time PA min/week	156	139	17	1531.0	1.99	0.047*	0.16	145	121	24	3004.5	2.94	0.003**	0.24
Total PA min/week	156	139	17	1231.0	0.28	0.778	0.02	145	121	24	1797.5	1.84	0.066	0.15
PA self-efficacy	-	-	-	-	-	-	-	135	114	21	1803.0	2.72	0.000***	0.23
PA enjoyment	-	-	-	-	-	-	-	140	117	23	1812.5	3.71	0.007**	0.31

- Not measured;

r^a^ effect size r = *z/*√N;

*p<0.050;

**p<0.010;

***p<0.001

Over the course of twelve months, between group comparisons showed that program dropouts continued to score significantly lower for LTPA (minutes/week) (*z* = 2.94, *p* = 0.003); for EQ-Index (*z* = 2.07, *p* = 0.039)–indicating that program dropouts more often reported (serious) complaints; for BMI (*z* = -2.17, *p* = 0.030)–indicating higher BMI among dropouts; for PA self-efficacy (*z* = 2.72, *p*<0.001); and PA enjoyment (*z* = 3.71, *p* = 0.007). Care consumption scored significantly higher among dropouts (*z* = -2.24, *p* = 0.025). No between group differences were found for overall PA and EQ-VAS (Table 6).

We did find evidence to support the hypothesis (H2) that CBHEPA participants performed better on physical activity and health-related quality of life outcomes than participants who dropped out of the CBHEPA program. The hypothesis (H2) was confirmed at T_1_ for perceived health and LTPA and at T_2_ for LTPA, and for variables relating to self-reported health complaints, BMI and care consumption. At T_2_ we also found significant differences for PA self-efficacy and PA enjoyment. For all but one indicators showing significant differences, effect sizes based on the *z-*scores (r) were small (r<0.20). PA enjoyment showed a medium effect size (r>0.30) (Table 6).

### Increase in leisure-time physical activity over time

Tables 7 and 8 summarise the results of the three-level growth models for LTPA. Table 6 presents the results of the analysis of LOG LTPA as outcome variable with perceived health (EQ-VAS) as health-related quality of life indicator. Starting with the null model (M0), stepwise correction was made for gender, age, ethnic origin and low educational level. Age proved to be the only factor improving the fit of the model, based on a significant decrease in REML (not reported in the table), but this effect disappeared when the SES factors were clustered (M1). Participation time, i.e. how long people participated in the CBHEPA program prior to the evaluation study, significantly improved the fit of the model (M2).

**Table 7:** Growth model for leisure-time physical activity (min/week) with perceived health (EQ-VAS).

Model	M0	M1	M2	M3	M4	M5	M6	M7	M8
		SES corrected^a^	Participation time	EQ-VAS	BMI	Total SoC3	PA self-efficacy	PA enjoyment	Group type
	Estimate (*s*.*e*.*)*	Estimate *(s*.*e*.*)*	Estimate *(s*.*e*.*)*	Estimate *(s*.*e*.*)*	Estimate *(s*.*e*.*)*	Estimate *(s*.*e*.*)*	Estimate *(s*.*e*.*)*	Estimate *(s*.*e*.*)*	Estimate *(s*.*e*.*)*
**Fixed part**									
Intercept	2.514 *(0*.*051)*	2.332 *(0*.*182)*	2.329 *(0*.*186)*	1.893 *(0*.*207)*	2.095 *(0*.*237)*	1.937 *(0*.*283)*	1.689 *(0*.*299)*	1.360 *(0*.*347)*	1.119 *(0*.*343)*
**Level 1: Intrapersonal**									
Time1	0.024 *(0*.*043)*	0.578 *(0*.*190)*	0.564 *(0*.*191)*	0.586 *(0*.*193)*	0.651 *(0*.*184)*	0.680 *(0*.*188)*	0.729 *(0*.*192)*	0.742 *(0*.*197)*	0.766 *(0*.*213)*
Time2	-0.032 *(0*.*046)*	-0.029 *(0*.*185)*	-0.051 *(0*.*186)*	0.017 *(0*.*184)*	0.130 *(0*.*177)*	0.127 *(0*.*180)*	0.133 *(0*.*183)*	0.134 *(0*.*183)*	0.142 *(0*.*201)*
Time3	*reference*								
Participation (no)	-0.460*** *(0*.*096)*	-1.330** *(0*.*464)*	-1.373** *(0*.*463)*	-1.391** *(0*.*459)*	-0.063 *(0*.*516)*	-0.201 *(0*.*534)*	-0.194 *(0*.*536)*	-0.093 *(0*.*543)*	-1.037 *(0*.*711)*
Time1*part.no	*All cases included*								
Time2*part.no	-0.015 *(0*.*136)*	-0.299* *(0*.*146)*	-0.362* *(0*.*152)*	-0.297* *(0*.*151)*	-0.107 *(0*.*143)*	-0.145 *(0*.*149)*	-0.143 *(0*.*150)*	-0.162 *(0*.*153)*	-0.426* *(0*.*194)*
Time3*part.no	*reference*								
**Level 2: Interpersonal**									
Gender (f)		0.099 *(0*.*146)*	0.083 *(0*.*149)*	0.086 *(0*.*144)*	0.052 *(0*.*143)*	0.061 *(0*.*145)*	0.040 *(0*.*146)*	0.056 *(0*.*149)*	0.041 *(0*.*148)*
Time1*Gend.(f)		-0.388** *(0*.*150)*	-0.373* *(0*.*151)*	-0364** *(0*.*151)*	-0.362* *(0*.*144)*	-0.374* (*0*.*146)*	-0.402** *(0*.*149)*	-0.410** *(0*.*152)*	-0.409** *(0*.*156)*
Time2*Gend.(f)		-0.194 (*0*.*142)*	-0.237 *(0*.*145)*	-0.213 *(0*.*143)*	-0.204 *(0*.*136)*	-0.207 *(0*.*138)*	-0.204 *(0*.*140)*	-0.194 *(0*.*140)*	-0.189 *(0*.*146)*
Time3*Gend (f)		*reference*							
Part.no*Gend (f)		-0.254 (*0*.*144)*	-0.220 *(0*.*273)*	-0.227 *(0*.*272)*	-0.218 *(0*.*252)*	-0.161 *(0*.*256)*	-0.176 *90*.*261)*	-0.232 *(0*.*263)*	-0.010 *(0*.*292)*
Ethnic origin		-0.050 *(0*.*098)*	-0.075 *(0*.*102)*	-0.046 *(0*.*099)*	-0.026 *(0*.*099)*	-0.011 *(0*.*102)*	-0.021 *(0*.*106)*	-0.012 *(0*.*110)*	0.002 *(0*.*119)*
Education low		0.162 *(0*.*085)*	0.162 *(0*.*086)*	0.171* *(0*.*086)*	0.163 *(0*.*083)*	0.156 *(0*.*084)*	0.179* *(0*.*086)*	0.198* *(0*.*089)*	0.215* *(0*.*091)*
Part.no* Educ. low (no)		0.130 *(0*.*203)*	0.108 *(0*.*202)*	0.122 *(0*.*205)*	-0.101 *(0*.*199)*	-0.018 *(0*.*216)*	-0.026 *(0*.*217)*	-0.051 *(0*.*230)*	0.040 *(0*.*244)*
Part.time < 3 months			0.087 *(0*.*067)*	0.081 *(0*.*061)*	0.126 *(0*.*064)*	0.120 *(0*.*066)*	0.075 *(0*.*070)*	0.102 *(0*.*074)*	0.192* *(0*.*082)*
Part.time 3–6 months			0.158 *(0*.*082)*	0.148 *(0*.*078)*	0.172* *(0*.*079)*	0.163* *(0*.*081)*	0.113 *(0*.*085)*	0.109 (*0*.*089)*	0.177 *(0*.*092)*
Part.time >3 months			*reference*						
*Health-related*									
EQ-VAS				0.005*** (*0*.*001)*	0.005*** *(0*.*001)*	0.004*** *(0*.*001)*	0.004** *(0*.*001)*	0.004** *(0*.*001)*	0.003* *(0*.*001)*
BMI					-0.007 *(0*.*004)*	-0.007 *(0*.*004)*	-0.005 *(0*.*004)*	-0.005 *(0*.*004)*	-0.004 *(0*.*004)*
Total SoC3						0.021 *(0*.*020)*	0.013 *(0*.*021)*	0.009 *(0*.*021)*	0.012 *(0*.*021)*
*Sport and PA*									
PA self-efficacy							0.014** *(0*.*005)*	0.010 *(0*.*005)*	0.013* *(0*.*005)*
PA enjoyment								0.011* *(0*.*004)*	0.011* *(0*.*004)*
**Level 3: PA group**									
PA group type 1									-0.433**(0.*156)*
PA group type 2									0.023 *(0*.*422)*
PA group type 3									-0.141 *(0*.*15)*
PA group type 4									*reference*
**Random part**									
Intercept (subj. = PA group)	0.016	0.007	0.012	0.006	0.012	0.013	0.014	0.019	0.011
Intercept (subj. = id*PA group)	0.057**	0.047	0.042	0.023	0.030	0.033	0.030	0.013	0.013
REML	676.78	595.70	570.85	554.27	512.99	510.88	501.41	484.33	483.53
*ΔREML(df)*^b^		*81*.*08(24)*	*24*.85(4)**	*16*.58(1)***	*41*.28(1)***	*2*.*11(1)*	*9*.47(1)**	*17*.08(1)***	*0*.*8(11)*

^a^ SES successively corrected for gender, age, ethnic origin, low education;

^b^ Assessment model improvement using ΔREML(df) and χ^2^-distribution;

*p<0.050;

**p<0.010;

*** *p*<0.001

**Table 8:** Growth model for leisure-time physical activity (min/week) with self-reported levels of health problems (EQ-Index).

Model	M0	M1	M2	M3	M4	M5T	M6	M7	M8
		SES corrected^a^	Participation time	EQ-VAS	BMI	Total SoC3	PA self-efficacy	PA enjoyment	Group type
	Estimate *(s*.*e*.*)*	Estimate *(s*.*e*.*)*	Estimate *(s*.*e*.*)*	Estimate *(s*.*e*.*)*	Estimate *(s*.*e*.*)*	Estimate *(s*.*e*.*)*	Estimate *(s*.*e*.*)*	Estimate *(s*.*e*.*)*	Estimate *(s*.*e*.*)*
**Fixed part**									
Intercept	2.514 *(0*.*051)*	2.332 *(0*.*182)*	2.329 *(0*.*186)*	2.127 *(0*.*198)*	2.222 *(0*.*229)*	2.089 *(0*.*273)*	1.818 *(0*.*293)*	1.1449 *(0*.*344)*	1.511 *(0*.*336)*
**Level 1: Intrapersonal**									
Time1	0.024 *(0*.*043)*	0.578 *(0*.*190)*	0.564 *(0*.*191)*	0.545 *(0*.*192)*	0.611 *(0*.*134)*	0.638 *(0*.*187)*	0.698 *(0*.*192)*	0.714 *(0*.*167)*	0.761 *(0*.*212)*
Time2	-0.032 *(0*.*046)*	-0.029 *(0*.*185)*	-0.051 *(0*.*186)*	-0.067 *(0*.*189)*	0.053 *(0*.*182)*	0.068 *(0*.*184)*	0.070 *(0*.*186)*	0.078 *(0*.*186)*	0.084 *(0*.*202)*
Time 3	*reference*								
Participation (no)	-0.460*** *(0*.*096)*	-1.330** *(0*.*464)*	-1.373** *(0*.*463)*	-1.447** *(0*.*461)*	-0.141 *(0*.*518)*	-0.227 *(0*.*532)*	-0.210 *(0*.*534)*	-0.113 *(0*.*542)*	-1.082 *(0*.*705)*
Time1*part.no									
Time2*part.no	-0.015 *(0*.*136)*	-0.299* *(0*.*146)*	-0.362* *(0*.*152)*	-0.369* *(0*.*153)*	-0.159 *(0*.*146)*	-0.180 *(0*.*150)*	-0.169 *(0*.*151)*	-0.171 *(0*.*154)*	-0.482* *(0*.*195)*
Time3*part.no	*reference*								
**Level 2: Interpersonal**									
Gender (f)		0.099 *(0*.*146)*	0.083 *(0*.*149)*	0.095 *(0*.*147)*	0.058 *(0*.*144)*	0.063 *(0*.*146)*	0.048 *(0*.*147)*	0.067 *(0*.*150)*	0.036 *(0*.*148)*
Time1*Gend(f)		-0.388** *(0*.*150)*	-0.373* *(0*.*151)*	-0.397** *(0*.*150)*	-0.392** *(0*.*144)*	-0.397** *(0*.*145)*	-0.431** *(0*.*148)*	-0.439** *(0*.*152)*	-0.423** *(0*.*155)*
Time2*Gend(f)		-0.194 *(0*.*142)*	-0.237 *(0*.*145)*	-0.250 *(0*.*147)*	-0.238 *(0*.*140)*	-0.228 *(0*.*141)*	-0.226 *(0*.*142)*	-0.215 *(0*.*142)*	-0.191 *(0*.*148)*
Time3*Gend(f)		*reference*							
Part. no*Gend(f)		-0.254 *(0*.*144)*	-0.220 *(0*.*273)*	-0.176 *(0*.*271)*	-0.159 (*0*.*252)*	-0.126 *(0*.*258)*	-0.152 *(0*.*260)*	-0.209 *(0*.*263)*	0.038 *(0*.*290)*
Ethnic origin		-0.050 *(0*.*098)*	-0.075 *(0*.*102)*	-0.072 *(0*.*101)*	-0.049 *(0*.*100)*	-0.036 *(0*.*102)*	-0.041 *(0*.*106)*	-0.031 *(0*.*111)*	-0.032 *(0*.*119)*
Education low		0.162 *(0*.*085)*	0.162 *(0*.*086)*	0.153 *(0*.*086)*	0.148 *(0*.*083)*	0.138 *(0*.*084)*	0.161 *(0*.*085)*	0.185* *(0*.*089)*	0.218* *(0*.*090)*
Part. no* Educ. low (no)		0.130 *(0*.*203)*	0.108 *(0*.*202)*	0.129 *(0*.*200)*	-0.091 *(0*.*195)*	-0.047 *(0*.*211)*	-0.054 *(0*.*212)*	-0.055 *(0*.*224)*	0.054 *(0*.*237)*
Part.time <3 months			0.087 *(0*.*067)*	0.106 *(0*.*065)*	0.149* *(0*.*065)*	0.141* *(0*.*067)*	0.093 *(0*.*071)*	0.112 *(0*.*075)*	0.208* *(0*.*082)*
Part.time 3–6 months			0.158 *(0*.*082)*	0.179* *(0*.*080)*	0.202* *(0*.*079)*	0.187* *(0*.*081)*	0.130 *(0*.*086)*	0.124 *(0*.*090)*	0.198 *(0*.*093)*
Part.time >3 months			*reference*						
*Health-related*									
EQ-Index				0.276** *(0*.*083)*	0.288**(0.*082)*	0.287** *(0*.*084)*	0.250** *(0*.*087)*	0.207* *(0*.*092)*	0.216* *(0*.*091)*
BMI					-0.006 (0.004)	-0.005 (0.004)	-0.004 (0.004)	-0.003 (0.004)	-0.002 (0.004)
Total SoC3						0.016 *(0*.*020)*	0.009 *(0*.*021)*	0.008 *(0*.*021)*	0.009 *(0*.*021)*
*Sport and PA*									
PA self-efficacy							0.014** *(0*.*006)*	0.012* *(0*.*005)*	0.014* *(0*.*005)*
PA enjoyment								0.011* *(0*.*004)*	0.010* *(0*.*004)*
**Level 3: PA group**									
PA group type 1									-0.461** *(0*.*154)*
PA group type 2									-0.031 *(0*.*419)*
PA group type 3									-0.105 *(0*.*154)*
PA group type 4									*reference*
***Random part***									
Intercept (subj. = PA group)	0.016	0.007	0.012	0.010	0.014	0.016	0.016	0.021	0.011
Intercept (subj. = id*PA group)	0.057**	0.047	0.042	0.041	0.041	0.040	0.038	0.023	0.022
REML	676.78	595.70	570.85	556.95	512.28	506.16	497.01	479.16	475.34
*ΔREML(df)*^b^		*81*.*08(24)*	*24*.85(4)**	*13*.9(1)**	*44*.67(1)***	*6*.12(1)*	*9*.15(1)**	*17*.85(1)***	*3*.*82(11)*

^a^ SES successively corrected for gender, age, ethnic origin, low education;

^b^ Assessment model improvement using ΔREML(df) and χ^2^-distribution;

*p<0.050;

**p<0.010;

*** *p*<0.001

Findings relating to the fixed effects at *intrapersonal* level in all models showed no significant within-subject differences in LOG LTPA at the three points of measurement. Time in interaction with program dropout in the full growth model (M8) showed a significant decrease in LOG LTPA among program dropouts compared to participants (*E* = -0.426, *p*< 0.050). After correction for SES variables, the change in LOG LTPA with perceived health showed a significant downward trend in the full growth model (M8) at T_1_ and T_2_ compared to baseline (*F*(2, 9.889, *p*<0.001). Differences between T_1_ and T_2_ were not significant.

Findings relating to the fixed effects at *interpersonal* level showed that women scored significantly lower at baseline on LOG LTPA (*p*<0.010) than men, but not in follow-up measurements. No significant differences were found between participants for age or ethnic origin. Findings relating to the full model (M8) for educational level suggested that LOG LTPA was significantly higher (*p*<0.050) among participants with higher educational levels, but that there was no significant difference in educational level between participants and program dropouts.

The time varying covariates in the successive models showed a significant improvement in the fit of the model at each step, except for SoC (M5), based on calculated differences in REML. This indicated that each covariate partly explained the variance in LOG LTPA. Perceived health (EQ-VAS) was significantly associated with higher levels of LOG LTPA in all models, whereas BMI and SoC were not. PA self-efficacy and PA enjoyment were also significantly associated with higher levels of LOG LTPA (*p*<0.050).

Findings relating to the fixed effects in the full model (M8) at *group* level showed that short CBHEPA programs (10–13 weeks) with multiple trainers, addressing gender homogeneous groups, were significantly associated with lower LOG LTPA levels whereas continuous CBHEPA programs with a single, known trainer, addressing gender-heterogeneous groups were not. Calculated effect sizes (Cohen’s d) for the different group types at the three points in times showed a medium effect at T_0_ (d = 0.51), and small effects at T_1_ (d = -0.12) and T_2_ (d = 0.07).

The variance of the intercepts between CBHEPA groups across the eight models was not significant, indicating that groups did not vary significantly in LTPA. The intercepts of participants (id) nested in PA groups, significant in the null model (M0), showed a gradual decline across the eight models. None of the included factors or covariates, however, significantly explained individual variance within groups (Table 7).

Table 8 presents the results of the parallel modelling of LOG LTPA as outcome variable with self-reported health complaints (EQ-Index) as health-related quality of life indicator. The estimation results for the models M0 to M2 were the same as reported in Table 6. Findings for modelling LOG LTPA and self-reported health complaints (EQ-Index) were similar to those for modelling LOG LTPA and perceived health (EQ-VAS). The full growth model (M8) for LOG LTPA with self-reported health complaints showed a significant downward trend at T_1_ and T_2_ compared to baseline (*F*(2,11.206), *p*<0.001). Differences between T_1_ and T_2_ were not significant.

The dataset used for the multilevel analysis of the growth model can be found in the supporting information (S2 Dataset).

Findings relating to the fixed effects at *intrapersonal* level in all models showed no significant within-subject differences in LOG LTPA at the three points of measurement. Time in interaction with program dropout in the full model (M8) showed a significant decrease in LOG LTPA in program dropouts compared to participants (*E* = -0.42, *p*< 0.050).

Findings relating to the fixed effects at *interpersonal* level showed that women scored significantly lower at baseline on LOG LTPA (*p*<0.010) than men, but not in follow-up measurements. No significant differences were found between participants for age or ethnic origin. Findings relating to the full model (M8) for differences in educational level suggested that LOG LTPA was significantly higher (*p*<0.050) among participants with higher educational levels, but that there was no significant difference in educational level between participants and program dropouts.

The time varying covariates in the successive models showed that lower scores on self-reported health complaints were significantly associated (*p*<0.050) with higher levels of LOG LTPA in all models, whereas BMI and SoC were not. PA self-efficacy and PA enjoyment were both significantly associated (*p*<0.050) with higher levels of LOG LTPA. SoC did, however, improve the fit of the model significantly (M5), indicating that SoC explained part of the variance in this model.

Findings relating to the fixed effects in the full model (M8) at *group* level were similar to those for the model LOG LTPA with perceived health: short CBHEPA programs (10–13 weeks) with multiple trainers, addressing gender homogeneous groups, significantly associated with lower LOG LTPA levels whereas continuous CBHEPA programs with a single, known trainer, addressing gender-heterogeneous groups were not. The development of the intercepts of CBHEPA groups across the eight models was similar to the pattern reported for the modelling of LOG LTPA and perceived health described above, as were the values for effect sizes (Cohen’s d) for the different group types at the three points in time.

In relation to the REML values in the parallel growth models for the two health-related quality of life indicators, the growth model for LOG LTPA with EQ-Index (REML = 475.34) showed a slightly better fit of model than the LOG LTPA with EQ-VAS (REML = 483.53). It is possible that perceived health is more strongly correlated with the other factors and covariates included in the model, such as BMI, SoC, PA self-efficacy and PA enjoyment, than EQ-Index.

We did not find evidence to confirm the hypothesis (H3) that participation in a CBHEPA program leads to an increase in its participants’ leisure-time physical activity levels over time. The positive association over time between health-related quality of life outcomes, physical activity self-efficacy and enjoyment, and leisure-time physical activity is, however, supported in the multilevel regression model.

## Discussion

In order to evaluate the effectiveness of group-based CBHEPA programs, the aim of this study was to assess whether or not CBHEPA programs contribute to increasing and maintaining physical activity in socially vulnerable groups over time. Based on a combination of statistical analyses, our findings do not univocally support the proposition that participation in a CBHEPA program leads to an increase in overall PA levels (quasi-RCT) or an increase in leisure-time PA at participant level after twelve months, as was hypothesised. The multilevel models showed significant positive associations between individual factors, such as higher education and being female, and leisure-time PA. Women scored significantly lower at baseline than men, but the gender-related difference in PA was not found in follow-up measurements. No significant differences were found between participants for age or, somewhat surprisingly, for ethnic origin. Health-related quality of life, PA self-efficacy and PA enjoyment were intrapersonal time varying covariates, significantly associated with higher levels of physical activity. Short CBHEPA programs (10–13 weeks) with multiple trainers were group-related factors associated with lower leisure-time PA over time compared to participants in on-going CBHEPA programs with a known, single trainer.

At twelve months, leisure-time PA levels of program dropouts were significantly lower compared to continuing participants, as were health-related quality of life, PA self-efficacy, and PA enjoyment outcomes. BMI and care consumption also scored significantly higher among dropouts. On the basis of our findings, it seems that intrapersonal time varying covariates are more relevant in explaining PA maintenance than interpersonal characteristics (e.g., gender, age or ethnic origin) or group level characteristics.

### Population reached

A first aspect relating to CBHEPA program effectiveness is whether or not the intended target population is reached. Socio-economic baseline data show that a majority of CBHEPA program participants have low educational levels (48.6%), low income (52.4%) and low employment rates (11%), compared to Dutch population data. Statistics Netherlands shows that 27% of the general population is lowly educated (no, or only primary, school), 10% have low income, and over 90% are employed [67–69]. Likewise, health-related quality of life indicators at baseline are lower than comparative research outcomes in Dutch population groups [58], and participants show a weaker SoC compared to other Dutch studies [70]. With an average BMI of 29.5 found in CBHEPA participants, the majority of the target group are overweight or obese. BMI data for the general population show 30% overweight (BMI 25–30) and 14% (BMI>30) obesity for women, and 47% overweight and 13% obesity for men [71]. BMI values require, however, a nuanced perspective since 32% of the CBHEPA participants are older than 65 years and over 60% are of non-Dutch origin, including a substantial number of participants from Asiatic backgrounds. The literature indicates that BMI is less appropriate as a measure for overweight in older and/or Asian population groups [72–74]. In terms of socio-economic and health-related quality of life outcomes at baseline, CBHEPA programs reach the intended target group (Table 9).

**Table 9:** Comparison of CBHEPA participants at baseline with Dutch population data.

Variable	CBHEPA participants	Dutch population	Source
***Socio-economic***			
Low education (%)	48.6	27	[67]
Low Income (%)	52.4	10	[68]
Employment %)	11.6	92	[69]
***Health-related Quality of Life***			
EQ index (-1–1) (mean)	0.72	0.89 (55–65 years)	[58]
EQ-VAS (0–100) (mean)	70.2	80.7 (55–65 years)	[58]
BMI >25 (%)			
women	75	44	[71]
men	82	60	
Sense of Coherence (%)	Strong: 14.3	Strong: 18.6	[70]
	Moderate: 51.4	Moderate: 60.3	
	Weak: 34.3	Weak: 21.1	
***Sport and physical activity***			
PA (*minutes/day*)	216	18–65 years: 202	[5]
		≥65 years: 130	

Overall PA levels, at an average of 216 minutes per day, are not low compared to Dutch trend analyses on sport and PA (Table 9). The latest trend report describes an increase from 169 to 202 minutes for Dutch adults (age 15–64) spent in PA during 2000–2011, mainly resulting from an increase in light and moderate intensity activities (in particular activities at work/school and at home). For older people (age 65 plus), there was an increase in PA from 100 to 130 minutes [5]. Our findings indicate that more than half of younger CBHEPA participants (< 65 years) were less active compared to the age-specific Dutch reference value (202 min/day) at all measurement points, whereas a majority of older CBHEPA participants (≥ 65 years) were more active compared to the age-specific Dutch reference value (130 min/day). These results suggest that CBHEPA programs reach both relatively inactive and active people. In terms of physical activity, it seems that, compared to the reference physical activity levels for adults, CBHEPA programs reach more inactive younger people (< 65 years) than inactive older people (≥ 65 years).

### Increase in PA levels over time?

A second aspect regarding CBHEPA program effectiveness is whether or not CBHEPA programs contribute to increasing and maintaining physical activity in socially vulnerable groups over time. Our findings do not show an increase over time. What is more, a significant decrease compared to baseline was observed. An American longitudinal multilevel study on community-based PA (neighbourhood walking) similarly reported a downward trend in PA over time [75]. There are several possible explanations for our findings.

First, for practical reasons of recruitment, participants were included at baseline only after the start of a CBHEPA program. Some programs had already existed for a number of years. At baseline, half of the participants had been active in the program for three months or more, resulting in the absence of genuine baseline data for PA and health-related quality of life.

Second, all data were assessed with self-report measures. For measuring PA, this is considered less reliable than an objective measure like an accelerometer [76]. We did not find, however, validated objective measurement instruments suitable for our target group, interpretable without additional self-report measures such as those collected with SQUASH. Self-report measures may also induce a question–behaviour effect: asking questions about a behaviour may change the behaviour in question [77, 78]. This usually leads to bias in a socially normative direction. During the repeated measurements, participants may have become also more experienced in answering the questions and at the same time may have developed a more realistic perspective on their own PA behaviour and health-related quality of life. A meta-analysis, though, found the question–behaviour effect on health-related behaviour to be rather small [79].

Third, the absence of an expected increase in leisure-time PA can be explained from a time allocation perspective. People tend to allocate only a certain amount of time daily to leisure time activities in general, and to PA or sport more particularly. This perspective is elaborated in the SLOTH model—a time-budget model incorporating Sleep, Leisure, Occupation, Transportation and Home-based activities—identifying possible economic factors of influence on individuals' choices about utilisation of time in relation to PA behaviour and maintenance [80, 81].

### PA maintenance in participants and program dropouts

Comparison of the multilevel models for the two health-related quality of life indicators reveals that perceived health (EQ-VAS) is possibly stronger correlated with other factors explaining leisure-time PA, such as BMI, SoC, PA self-efficacy and PA enjoyment, than self-reported health complaints (EQ-Index). Both models, however, offer solid indications that PA maintenance is strongly related to health-related quality of life on the one hand, and PA self-efficacy and PA enjoyment on the other. These findings are in line with other studies showing evidence for the interrelatedness of health and PA behaviour [8] and the role of (post) motivational factors in PA maintenance [29, 35, 36].

Our findings indicate that leisure-time PA, health-related quality of life indicators, BMI, PA self-efficacy, and PA enjoyment score worse among program dropouts. One explanation is that health impairments are the main reason given for participants to quit the program. Dutch CBHEPA programs targeting socially vulnerable groups may, therefore, need to focus on actions to prevent lapses resulting from health complaints, and help people cope with risk situations for lapses, thus enforcing program adherence and PA maintenance. [27, 82].

### Group level characteristics

Our findings show that group effects do have an impact on (leisure-time) PA behaviour and maintenance. Short CBHEPA programs (10–13 weeks) with multiple trainers, addressing gender-homogeneous groups, were significantly associated with lower leisure-time PA levels than on-going CBHEPA programs with a single, known trainer, addressing gender-heterogeneous groups. The observed decline in effect sizes over time may be a result of the fact that participants of short-term programs may have been less represented in the follow up measurements. The findings from this quantitative multilevel study are, however, supported by several qualitative studies on group effects, indicating that group dynamics, group composition and social support, and exercise trainer characteristics contribute substantially to effective PA programs [38, 39, 83, 84].

### Methodological issues

Our findings should be interpreted in the context of several strengths and limitations. A first strength of our study is that we evaluated on-going field practice, rather than conducting an experimental setup, to investigate the determinants of PA behaviour and maintenance in socially vulnerable groups. Creating controlled experimental conditions are of limited value to contribute substantially to a (practice based) body of evidence needed to understand what works for whom in CBHEPA programs [45, 85, 86]. For example, the use of adequate control groups can be problematic, since matching for non-observable differences such as initial motivation, is not easily done. Therefore, our study locked onto natural experiments—the CBHEPA programs—by design. Natural experiments have an important contribution to make to the health and PA inequalities agenda, including assessment of effective interventions, an area which is acknowledged as lacking an evidence-base [87]. In our experience, the sequential cohort design, in which the intervention effects are measured repeatedly using the T_0_ measurements as point of reference, proves a feasible approach. In addition, it offers the possibility to compare between cohorts, i.e. in our case between program adherents and starters [44].

A second strength is the use of multilevel modelling in this study to monitor physical activity development over time in socially vulnerable groups. Multilevel analysis and repeated measurements are not often used to assess CBHEPA program effectiveness, and our use of these techniques adds to the commonly used individual-level research design paradigm [25, 75]. The inclusion of intra-individual factors (covariates), as well as inter-individual and group-level factors contributes to the strength of the study.

A third strength is the longitudinal nature of the study, addressing a critical need for data on patterns of PA behaviour and maintenance and how these may change over time. As some researchers indicate, a multilevel perspective allows researchers to identify significant and potentially modifiable factors, and this in turn can inform policy changes and facilitate the design of interventions to change health and PA behaviour at societal level [25, 88].

Limitations to our study relate first to the limited number of determinants of potential influences on PA behaviour in socially vulnerable groups, included in our data collection. Given our target group, we were challenged to balance our information needs and the target group’s responsive capacity and competences. Questionnaire use can be difficult in socially vulnerable groups. Lack of health literacy, lack of basic skills in reading and writing and different beliefs about health concepts across cultures may lead to difficulties in understanding and interpreting the questions [47, 89], eventually leading to non-response [88]. Alternatives, however, such as translations, working with images or digital devices, suffer similar limitations [90, 91]. During our study, we did experience a number of these barriers in data collection. Steps were taken to deal with response difficulties by limiting the number of questions reducing the number of indicators, or by choosing restricted scales, such as the SoC three-item instead of the SoC thirteen-item instrument [51]. It thus forced us to limit ourselves to collect information about the most important explanatory factors for PA behaviour and maintenance found in CBHEPA programs, such as health-related quality of life, PA self-efficacy and PA enjoyment. Using a personalised data collection strategy [47], advocated by CBHEPA professionals and practitioners, was successful in reaching out to and inclusion of a satisfactory number of participants. We cannot, however, rule out the fact that other contextual influences (e.g., family situation, community or neighbourhood), not included in our study, may also have been important in explaining PA behaviour and maintenance. In particular, neighbourhood factors have been found to play a significant role in PA and other health behaviours [92].

A second limitation relates to the validity of the standardised instruments compiled in our questionnaire, when using them in our target group. The SQUASH instrument in particular was perceived as complicated by participants, because of its number of items and the seven-day recall structure. Moreover, participants had (to be able) to reflect on their PA behaviour and make time calculations. To tackle this issue, we monitored the data collection procedure closely throughout our study by making observational notes, and by reviewing the forms for missing items, illegible handwriting, inadequate answers and logical inconsistencies among responses after each data collection session. Errors thus identified were resolved by checking back with the participant, the trainer or the assistant [93].

A third limitation of our study relates to potential sources for bias. Recruitment of participants, done in collaboration with practice and on voluntary basis, may have suffered from a selection bias. Only people willing to participate were included. It also resulted in a lack of genuine baseline data, since the researcher could not contact participants before PA groups had started. Similarly, in comparing participants and program dropouts, a selection bias may have plaid a role, as we relied on people willing to fill out questionnaires after having quit the CBHEPA program.

The survey settings, usually the PA group setting at the sports venue, may have influenced people’s responses. Using the sport venue, however, as communal factor throughout the study has contributed to minimising this bias. In addition, using the multilevel analysis helped to correct for possible interdependencies in responses within groups.

### Future research

Over the past decade, the ecological perspective has gained ground as a new paradigm in research on PA behaviour and maintenance [19, 94–96]. It is to be expected that this will lead to more transdisciplinary research [97] and the use of hierarchical data structures and multilevel statistical procedures [25, 75, 88]. What our study shows is that studying socially vulnerable groups from the perspective of PA and health inequalities, applying multilevel modelling, still suffers from highly abstracted social concepts to make them measurable and interpretable. Concise, interpretative mixed-method research, combining quantitative and qualitative research data in one study, could help identify the contextualised explanatory factors for particular groups in more detail, hence improving the accuracy of statistical procedures [98].

## Conclusion

Dutch CBHEPA programs reach relatively socially vulnerable, but not necessarily inactive, groups, in terms of socio-economic and health–related quality of life outcomes. No increase in leisure-time physical activity behaviour could be observed over time, but health-related quality of life, self-efficacy and enjoyment were found to contribute to physical activity maintenance. A decrease became manifest in physical activity as well as in health-related quality of life-related outcomes among dropouts. Our findings suggest that CBHEPA programs contribute to physical activity maintenance in socially vulnerable groups. These programs should, therefore, be valued for their potential in encouraging program adherence, rather than being made accountable for increasing physical activity.

## Supporting Information

S1 Dataset SPSS22 Working set Group Means comparison CoM survey. (SAV)

S2 Dataset SPSS22 Mixed Model analysis CoM survey. (SAV)

S1 Table Overview of variables measured over time in relation to PA behaviour. (PDF)

S1 Text Multilevel analysis using SPSS 22 Mixed Model: Example of the syntax. (PDF)

## Abbreviations

BMI: Body mass index
CBHEPA: Community-based health enhancing physical activity
CoM: Communities on the Move
EQ-5D-3L: Five-dimensions, three-level Euro Quality-of-Life questionnaire
EQ-Index: Index based on EQ-5D-3L outcomes (Dutch time-trade-off value set)
EQ-VAS: Visual analogue scale for self-rating health-related quality of life
LOG LTPA: Log transformed leisure-time physical activity
LTPA: Leisure-time physical activity
NISB: Netherlands Institute for Sport and Physical Activity
PA: Physical activity
PACES: Physical activity enjoyment scale
REML: Restricted maximum likelihood
RCT: Randomised controlled trial
SES: Socio-economic status
SoC: Sense of Coherence
SCT: Social cognitive theory
SQUASH: Short Questionnaire to Assess Health-enhancing Physical Activity
QoL: Quality of Life
WHO: World Health Organisation
ZonMw: Netherlands Organisation for Health Research and Development
