# Supplementary material for: Health-Related Quality of Life, Self-Efficacy and Enjoyment Keep the Socially Vulnerable Physically Active in Community-Based Physical Activity Programs: A Sequential Cohort Study
Source: PLoS One. 2016 Feb 24;11(2):e0150025. doi: 10.1371/journal.pone.0150025 (PMC4766301; doi:10.1371/journal.pone.0150025)
Supplement: S1 Table — (PDF) [file pone.0150025.s003.pdf]

Supporting information S1

Table Overview of variables measured over time in relation to PA behaviour

Variable	Mode of measurement	# Items	Scale	Reference	T_0	T_1	T_2
**Personal**							
Sex	What is your gender?	1	2-point scale: 0= female; 1=male	[48]	x		
Age	What is your birth year?	1	continuous	[48]	x		
Ethnic origin	What is your country of birth?<br>If not born in the Netherlands, how long do you live in the Netherlands (years)?	1<br>1	Open ended<br>continuous	[48]	x		
**Socio-economic**							
Income	What is your net monthly household income?	1	5-point scale: =1000 euro or less…5=I don't know/not willing to say	[48]	x		
Education	What is your highest certified educational level?	1	3-point scale: 1=no/primary; 2=secondary; 3=higher/university	[48]	x		
**Health-related quality of life**							
Personal motivation	What was your main reason to join the CBHEPA program?	1	open-ended		x		
Health related Quality of Life	EuroQoL 5D-3L	5	3-point scale: 1=no complaints; 2= moderate complaints; 3= severe complaints	[56]	x	x	x
	EQ-Index, computed based on EuroQoL 5D-3L	1	Continuous, ranging from -1 to 1	[58]	x	x	x
	Visual analogue scale (EQ-VAS)	1	0 - 100 rating scale	[56]	x	x	x
Sense of Coherence (SoC3)	'Do you usually see solutions to problems and difficulties that other people find hopeless?' (*manageability*)<br>'Do you usually feel that your daily life is a source of personal satisfaction?' (*meaningfulness*)<br>'Do you usually feel that the things that happen to you in your daily life are hard to understand?'(*comprehensibility*).	3	3-point scale:3=yes, usually; 2= yes sometimes; 1=no<br>*Cronbach;'s α:0.43*	[51, 52, 53]	x		
**Physical activity**							
SQUASH modified	Work related physical activity	2	Continuous (minutes/week)	[54, 55]	x	x	x
	Work related commuting	3	Continuous (minutes/week)		x	x	x
	Household related physical activity	2	Continuous (minutes/week)		x	x	x
	Leisure time physical activity (walking, cycling, gardening, do-it-yourself activity)	4	Continuous (minutes/week)		x	x	x
	Do you do any additional sport?	4	Continuous (minutes/week)		x	x	x
Sport participation	Are you a (former) member of a sport club?	1	3-point scale, 1=yes; 2= no, but I used to; 3= No, never		x		
CBHEPA Program participation	Since when do you participate?	1	3-pointscale: 1=less than 3 months; 2=3-6 months;3=longer than 6 months		x		
	How many times a week do you participate?	1	4-pointscale: 1=less than 1x week; 2=1xweek …; 3=over 2xweek		x		
PA self-efficacy	"I am confident that I am able to participate in the PA program during the coming months."<br>(…when I am tired …: when I have pain/complaints..; when I return holiday; … when the program stops…).	5	5-point scale: 5=strongly agree; …<br>1= strongly disagree<br>*Cronbach;'s α:0.70*	[60]	x		x
PA Enjoyment	Short Physical Activity Enjoyment Scale (PACES) "when I exercise or sport, I enjoy it/I feel bored/ hate it/is it fun/feel good physically while doing it."	9	5-point scale: 5=strongly agree; …<br>1= strongly disagree<br>*Cronbach;'s α:0.73*	[61]	x		x
