# Supplementary material for: Health-Related Quality of Life, Self-Efficacy and Enjoyment Keep the Socially Vulnerable Physically Active in Community-Based Physical Activity Programs: A Sequential Cohort Study
Source: PLoS One. 2016 Feb 24;11(2):e0150025. doi: 10.1371/journal.pone.0150025 (PMC4766301; doi:10.1371/journal.pone.0150025)
Supplement: S1 Text — (PDF) [file pone.0150025.s004.pdf]

Supporting information S1 Text

Multilevel analysis using SPSS 22 Mixed Model: Example of the syntax

NULL MODEL (M0)

```
MIXED LOG_Tot_LTPA BY Time Participation
 /CRITERIA=CIN(95) MXITER(100) MXSTEP(10) SCORING(1) SINGULAR(0.000000000001)
HCONVERGE(0,
 ABSOLUTE) LCONVERGE(0, ABSOLUTE) PCONVERGE(0.000001, ABSOLUTE)
 /FIXED= Time Participation Time * Participation | SSTYPE(3)
 /METHOD=REML
 /PRINT=G SOLUTION TESTCOV
 /RANDOM=INTERCEPT | SUBJECT(BG) COVTYPE(ID)
 /RANDOM=INTERCEPT | SUBJECT(BG*id) COVTYPE(ID)
 /REPEATED= Time | SUBJECT(BG*id) COVTYPE(AR1)
 /EMMEANS=TABLES(Time) COMPARE ADJ(LSD)
 /EMMEANS=TABLES(Participation) COMPARE ADJ(LSD).
```

FULL MODEL (M8)

```
MIXED LOG_Tot_LTPA BY Time Participation Gender Age_CAT Dutch_Origin Low_Educ DuurRC
PA_Group WITH
 EQ_Index BMI Tot_SoC3RC SETOTR Tot_PA_Enjoy
 /CRITERIA=CIN(95) MXITER(100) MXSTEP(10) SCORING(1) SINGULAR(0.000000000001)
HCONVERGE(0,
 ABSOLUTE) LCONVERGE(0, ABSOLUTE) PCONVERGE(0.000001, ABSOLUTE)
 /FIXED= Time Participation Time*Participation Gender Time*Gender Participation * Gender
 Age_CAT Time * Age_CAT Participation * Age_CAT Dutch_Origin Time * Dutch_Origin Participation *
Dutch_Origin Low_Educ Time * Low_Educ
 Participation * Low_Educ DuurRC EQ_Index BMI Tot_SoC3RC SETOTR Tot_PA_Enjoy PA_Group
Time * PA_Group
 Participation * PA_Group | SSTYPE(3)
 /METHOD=REML
 /PRINT=G SOLUTION TESTCOV
 /RANDOM=INTERCEPT | SUBJECT(BG) COVTYPE(ID)
 /RANDOM=INTERCEPT | SUBJECT(BG*id) COVTYPE(ID)
 /REPEATED=Time | SUBJECT(BG*id) COVTYPE(AR1)
 /EMMEANS=TABLES(Time) COMPARE ADJ(LSD)
 /EMMEANS=TABLES(Participation ) COMPARE ADJ(LSD)
/EMMEANS=TABLES(Time * Gender )
```
